# Deterioration Level Estimation Based on Convolutional Neural Network Using Confidence-Aware Attention Mechanism for Infrastructure Inspection

**DOI:** 10.3390/s22010382

**Published:** 2022-01-05

**Authors:** Naoki Ogawa, Keisuke Maeda, Takahiro Ogawa, Miki Haseyama

**Affiliations:** 1 Graduate School of Information Science and Technology, Hokkaido University, N-14, W-9, Kita-ku, Sapporo 060-0814, Hokkaido, Japan; 2 Office of Institutional Research, Hokkaido University, N-8, W-5, Kita-ku, Sapporo 060-0808, Hokkaido, Japan; maeda@lmd.ist.hokudai.ac.jp; 3 Faculty of Information Science and Technology, Hokkaido University, N-14, W-9, Kita-ku, Sapporo 060-0814, Hokkaido, Japan; ogawa@lmd.ist.hokudai.ac.jp (T.O.); miki@ist.hokudai.ac.jp (M.H.)

**Keywords:** deterioration level estimation, convolutional neural network, attention map, confidence, infrastructure inspection

## Abstract

This paper presents deterioration level estimation based on convolutional neural networks using a confidence-aware attention mechanism for infrastructure inspection. Spatial attention mechanisms try to highlight the important regions in feature maps for estimation by using an attention map. The attention mechanism using an effective attention map can improve feature maps. However, the conventional attention mechanisms have a problem as they fail to highlight important regions for estimation when an ineffective attention map is mistakenly used. To solve the above problem, this paper introduces the confidence-aware attention mechanism that reduces the effect of ineffective attention maps by considering the confidence corresponding to the attention map. The confidence is calculated from the entropy of the estimated class probabilities when generating the attention map. Because the proposed method can effectively utilize the attention map by considering the confidence, it can focus more on the important regions in the final estimation. This is the most significant contribution of this paper. The experimental results using images from actual infrastructure inspections confirm the performance improvement of the proposed method in estimating the deterioration level.

## 1. Introduction

The number of aging infrastructures is increasing around the world [1,2], and techniques to support engineers in maintaining infrastructures efficiently are required. For their maintenance, engineers usually perform visual inspections of distresses that occur in infrastructures and record the progress of distresses as deterioration levels [3]. In particular, engineers first capture distress images in on-site inspections and determine the deterioration levels at conferences after the inspections using the distress images. To support such inspections, methods for estimating the deterioration level using the distress images [4] and automatic distress image capturing by robots [5] have been proposed. Notably, several skilled engineers need to make reliable determinations because the determination of deterioration level in the conferences is the final judgment. Their burden is high because of the large number of distress images to be examined at the conference. Therefore, reducing the burden on the engineers by accurately estimating the deterioration level based on machine learning using distress images is necessary.

**Figure 1:**
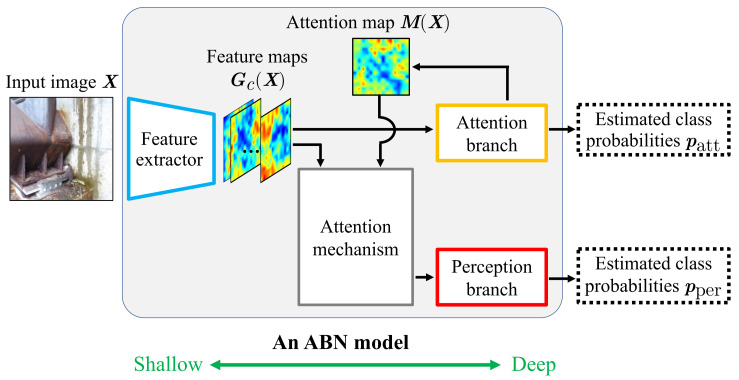
Overview of ABN. During the estimation, the feature extractor first outputs feature maps by using the input image. Next, the attention branch generates an attention map using the feature maps. Then the attention mechanism highlights important regions in the feature maps using the attention map. Finally, the perception branch performs the final estimation using the feature maps obtained from the attention mechanism.

Based on convolutional neural networks (CNNs) [6], the performance in various image recognition tasks has been improved [7]. However, general CNNs for estimation tasks output only the estimated class probabilities and do not explain the estimation results. Because misjudgment of the deterioration levels may endanger people using the infrastructure, high reliability is required. When engineers refer to the results of deterioration level estimation, trusting the estimation results that have unknown reasons is difficult. Therefore, methods that can explain the reasons for the estimation results are required. Recently, several methods have been proposed to improve estimation performance and interpretability using attention, which enables focusing on important features [8,9]. In image recognition, an attention branch network (ABN), which uses an attention map generated during the CNN-based estimation to improve estimation performance and explain the estimation results, has been proposed [10]. ABN has been used in various fields, such as self-driving, deterioration level estimation, and control of household service robots [11,12,13,14]. Figure 1 shows an overview of ABN. The ABN model has a feature extractor in the shallow part near the input and two branches called an attention branch and a perception branch in the deep part near the output. The attention branch performs estimation before the perception branch using feature maps output from the feature extractor. It also generates an attention map that represents the regions of interest in the estimation. The generated attention map is used to highlight important regions in the feature maps in the attention mechanism. The perception branch performs the final estimation using the feature maps that are output from the attention mechanism. The attention map is a spatial annotation that enables focusing on the important regions in the feature maps. However, the estimation performance is degraded when the actual regions of distresses are different from the regions highlighted by the attention map [15]. The influence of the attention map that does not highlight important regions needs to be reduced based on the reliability of whether the regions highlighted by the attention map are actual regions of distresses. Recently, several studies have reported on the use of attention by considering its reliability [16,17]. For example, the literature [16] has reported the effectiveness of using only useful attention by considering the relationship between attention and query in image captioning. Moreover, it has been reported in [17] that it is effective to use the attention that introduces the concept of uncertainty using a Bayesian framework for disease risk prediction. Moreover, the literature [18] has reported the effectiveness of learning using data with “noisy labels”, which are labels with uncertain reliability, while considering the confidence in the labels. Therefore, in the ABN-based estimation, it can be more effective to reduce the influence of highlighted regions that are irrelevant to the actual distress regions. In other words, it is expected that the performance of the deterioration level estimation can be improved by preferentially utilizing an attention map that highlights regions related to the distress.

To investigate the reliability of the attention map, we focus on the confidence in the estimated class probabilities corresponding to the attention map. In the ABN-based estimation, the attention branch outputs the estimated class probabilities and the corresponding attention map representing the regions of interest. The confidence in the estimated class probabilities that are output by the attention branch can be used to investigate the reliability of the attention map. The entropy calculated from the estimated class probabilities has been widely used to estimate the uncertainty of the estimated class probabilities [19]. The smaller the uncertainty, the higher the confidence in the estimated class probabilities. An attention map corresponding to the estimated class probabilities with low confidence is expected to contain a lot of noise, i.e., the attention map does not accurately highlight the important regions for the estimation. Therefore, it is possible to calculate the confidence in the attention map from the entropy of the estimated class probabilities. The performance of the deterioration level estimation improves significantly by controlling the influence of the attention map on the feature maps according to the above confidence.

**Figure 2:**
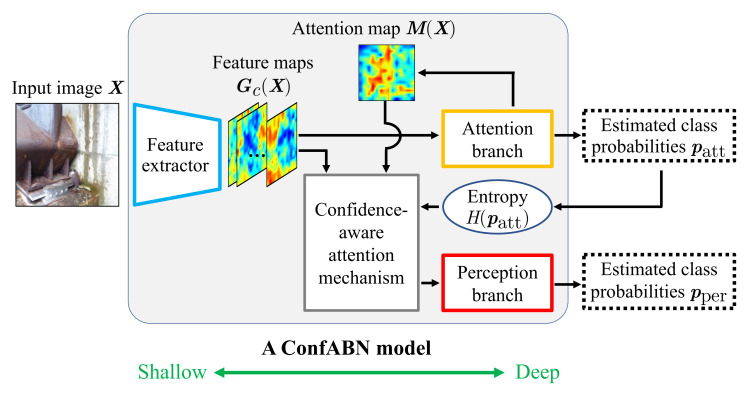
Overview of ConfABN. The feature extractor and attention branch of ConfABN output the feature maps and generate the attention map, respectively, in the same manner as the original ABN. The difference between ConfABN and ABN is that ConfABN uses a confidence-aware attention mechanism, which is an improved attention mechanism. Specifically, the confidence-aware attention mechanism can consider the confidence in the attention map by utilizing the entropy calculated from the estimated class probabilities of the attention branch. Consequently, the feature maps are strongly influenced by the effective attention map and weakly influenced by the ineffective attention map. Thus, ConfABN improves the performance of the final estimation in the perception branch.

In this paper, deterioration level estimation based on confidence-aware ABN (ConfABN), which can control the influence of an attention map on feature maps according to the confidence, is proposed for infrastructure inspection. Figure 2 shows an overview of ConfABN. We improve a conventional attention mechanism in ABN so that an attention map with high confidence has a strong influence on the feature maps. Specifically, we input the entropy-based confidence calculated from the estimated class probabilities of the attention branch to our attention mechanism in addition to the original inputs. In our attention mechanism, feature maps are multiplied by an attention map weighted based on the confidence so that a reliable attention map can be used strongly and an unreliable attention map can be used weakly. The above attention mechanism that can consider the confidence is called the confidence-aware attention mechanism, and it is the greatest contribution of this paper. The perception branch performs the final estimation using the feature maps obtained from the confidence-aware attention mechanism. Consequently, accurate deterioration level estimation can be realized by using the attention map considering its confidence. ConfABN has higher visual explainability for estimation results by presenting spatial attention maps than methods that employ a channel attention mechanism [20] to determine the attention of each channel of the feature maps, such as SENet [9]. Therefore, ConfABN is suitable for supporting the inspection of infrastructures that need high reliability. Furthermore, since ConfABN can provide confidence in the attention map of the estimation results, it achieves higher explainability for the output than previous attention-based methods, such as ABN [10].

The remainder of the paper is organized as follows. In Section 2, the estimation of deterioration level based on ConfABN is described. Experimental results are described in Section 3 to verify the effectiveness of the proposed method. Finally, Section 4 presents the conclusions.

## 2. Deterioration Level Estimation Based on Confabn

In this section, the estimation of deterioration level based on ConfABN is explained. As shown in Figure 2, ConfABN consists of three modules: a feature extractor, an attention branch, and a perception branch. The feature extractor is constructed with convolutional layers and calculates feature maps $G_{c}\left(X\right)$ (c=1,2,…,C;C being the number of channels of the feature maps) from an input distress image X. By using the feature maps $G_{c}\left(X\right)$, the attention branch outputs an attention map M(X) and estimated class probabilities $p_{\mathrm{att}}$ used to train the ConfABN model. Then we calculate improved feature maps $G_{c}^{\prime }\left(X\right)$ based on the confidence-aware attention mechanism from the feature maps $G_{c}\left(X\right)$ and the attention map M(X). The perception branch outputs the final estimated class probabilities $p_{\mathrm{per}}$ using the improved feature maps $G_{c}^{\prime }\left(X\right)$. It is worth noting that the feature extractor, attention branch, and perception branch are constructed by partitioning the CNN model commonly used in general classification tasks, such as ResNet [21], which is described in detail in [10]. In Section 2.1, Section 2.2 and Section 2.3, we explain the attention branch, confidence-aware attention mechanism, and perception branch, respectively. Furthermore, Section 2.4 describes the training of ConfABN.

### 2.1. Attention Branch

The feature maps $G_{c}\left(X\right)$ calculated by the feature extractor are input into the attention branch. The attention branch has multiple convolutional layers on the input side. On the output side, the attention branch has a global average pooling layer for calculating class probabilities $p_{\mathrm{att}}$ and a convolutional layer for calculating the attention map M(X). The dimension of the output of the global average pooling layer is the number of classes. Since this layer has the softmax function as the activation function, it can output the estimated class probabilities $p_{\mathrm{att}}$ in the attention branch. These estimated class probabilities $p_{\mathrm{att}}$ are used for the confidence-aware attention mechanism, as described in detail in Section 2.2. Furthermore, $p_{\mathrm{att}}$ are used for the training of the attention branch, as described in Section 2.4. Notably, the layer for calculating the attention map M(X) is the 1×1×1 convolutional layer, which has a sigmoid function as the activation function. Here, the 1×1×1 convolutional layer has a 1×1 kernel, and the number of channels of its output is 1. The obtained attention map M(X) can be used in the confidence-aware attention mechanism to improve feature maps $G_{c}\left(X\right)$ considering the confidence in the estimated class probabilities $p_{\mathrm{att}}$.

The generation of the attention map in the attention branch is designed with reference to class activation mapping (CAM) [22]. It is a method for visualizing the regions of interest that a CNN model focuses on during the test phase. As explained in detail in [10], CAM cannot generate the attention map during training because it uses feature maps and the weights of fully connected layers obtained after training. The attention branch uses a 1×1×1 convolutional layer to compute the attention map in a feedforward process and can output the attention map even during training. However, in the early epochs of training, many ineffective attention maps are likely to be generated since the parameters of the model are not sufficiently optimized. The use of such an ineffective attention map in the attention mechanism is a problem of the original ABN. As presented in the next subsection, ConfABN enhances the usefulness of the attention branch by considering the confidence in the attention map during the training.

### 2.2. Confidence-Aware Attention Mechanism

The confidence-aware attention mechanism improves the feature maps $G_{c}\left(X\right)$ using the attention map M(X) and the estimated class probabilities $p_{\mathrm{att}}$ from the attention branch. In the attention mechanism of the original ABN, the attention map M(X) is applied to the feature maps $G_{c}\left(X\right)$ using the following equation to calculate the improved feature maps $G_{c}^{\prime }\left(X\right)$:

(1) $$\begin{array}{c} G_{c}^{\prime }\left(X\right)=\left\{1+M\left(X\right)\right\}⊙G_{c}\left(X\right), \end{array}$$

where ⊙ denotes the Hadamard product. The matrix whose elements are 1, and its size is equal to M(X) is denoted by 1. However, in the confidence-aware attention mechanism, the improved feature maps $G_{c}^{\prime }\left(X\right)$ are calculated by applying the attention map M(X) to feature maps $G_{c}\left(X\right)$ as follows:

(2) $$\begin{array}{cc} G_{c}^{\prime }\left(X\right) & =\left\{1+t\cdot M\left(X\right)+\left(1-t\right)\cdot 1\right\}⊙G_{c}\left(X\right) \end{array}$$

(3) $$\begin{array}{cc} & =\left\{2\cdot 1+t\cdot \left(M\left(X\right)-1\right)\right\}⊙G_{c}\left(X\right), \end{array}$$

where

(4) $$\begin{array}{cc} t & =1-\frac{H(p_{\mathrm{att}})}{\max\left(H(p_{\mathrm{att}})\right)}\;, \end{array}$$

where *t* (0≦t≦1) denotes the confidence calculated from the entropy $H(p_{\mathrm{att}})$. Note that $H(p_{\mathrm{att}})$ is calculated using the class probabilities $p_{\mathrm{att}}\;(=\left[p_{\mathrm{att}}^{1},\ldots ,p_{\mathrm{att}}^{N}\right]$; *N* as the number of the deterioration levels) output from the attention branch as follows:

(5) $$\begin{array}{c} H\left(p_{\mathrm{att}}\right)=-\sum_{n=1}^{N}p_{\mathrm{att}}^{n}\log p_{\mathrm{att}}^{n}\;. \end{array}$$

The entropy becomes maximum when the probability is uniformly distributed, and its value is a constant that depends only on *N*. A large value of the entropy $H(p_{\mathrm{att}})$ reduces the confidence *t*; thus, the coefficient of the attention map M(X) becomes smaller. Therefore, an attention map that seems ineffective due to low confidence will have a smaller influence on feature maps. Moreover, it is possible to use a strong attention map that seems effective due to high confidence. Consequently, the confidence-aware attention mechanism can consider the effectiveness of the attention map, and it improves the performance in the perception branch.

The confidence-aware attention mechanism can reduce the negative effects of ineffective attention maps that are likely to be generated in the early training process. Furthermore, our attention mechanism can prevent the negative effects of ineffective attention maps in the test phase. Section 3.2 demonstrates that the distribution of confidence has many small values in the early stages of training, and ineffective attention maps with small confidence are generated in the test phase.

### 2.3. Perception Branch

The perception branch calculates the final estimated class probabilities $p_{\mathrm{per}}$ using the improved feature maps $G_{c}^{\prime }\left(X\right)$ as input. Specifically, in the perception branch, the improved feature maps $G_{c}^{\prime }\left(X\right)$ are first propagated through multiple convolutional layers. Then, the output of the last convolutional layer is input into a global average pooling layer to obtain a feature vector. By inputting the feature vector into a fully connected layer with the softmax function as the activation function, the final estimated class probabilities $p_{\mathrm{per}}$ for the deterioration level are output. Consequently, ConfABN achieves an accurate deterioration level estimation using the feature maps improved by the confidence-aware attention mechanism.

### 2.4. Training of ConfABN

ConfABN is trained in an end-to-end manner using a loss function *L* calculated from the estimated class probabilities $p_{\mathrm{att}}$ and $p_{\mathrm{per}}$. *L* is defined by the following equation:

(6) $$\begin{array}{cc} L & =L_{\mathrm{att}}\left(p_{\mathrm{att}}\right)+\alpha \cdot L_{\mathrm{per}}\left(p_{\mathrm{per}}\right), \end{array}$$

(7) $$\begin{array}{cc} L_{\mathrm{att}} & =-\sum_{n=1}^{N}q^{n}\log p_{\mathrm{att}}^{n}\;, \end{array}$$

(8) $$\begin{array}{cc} L_{\mathrm{per}} & =-\sum_{n=1}^{N}q^{n}\log p_{\mathrm{per}}^{n}\;, \end{array}$$

where $L_{\mathrm{att}}\left(p_{\mathrm{att}}\right)$ and $L_{\mathrm{per}}\left(p_{\mathrm{per}}\right)$ are the losses calculated by inputting $p_{\mathrm{att}}$ and $p_{\mathrm{per}}$ into the cross-entropy loss function, respectively. $q^{n}$ is 1 if class *n* is equal to the ground truth and 0 otherwise; α is a hyperparameter for adjusting the influence of $L_{\mathrm{per}}\left(p_{\mathrm{per}}\right)$. The end-to-end training of the feature extractor, attention branch, and perception branch can be performed using the loss function *L*. In other words, training ConfABN with *L* realizes simultaneous optimization of the parameters of the model for the attention map generation and final deterioration level estimation.

## 3. Experimental Results

In this section, we present the experimental results to verify the effectiveness of the proposed method. The experimental settings are explained in Section 3.1; performance evaluation and discussions are explained in Section 3.2.

### 3.1. Experimental Setting

**Figure 3:**
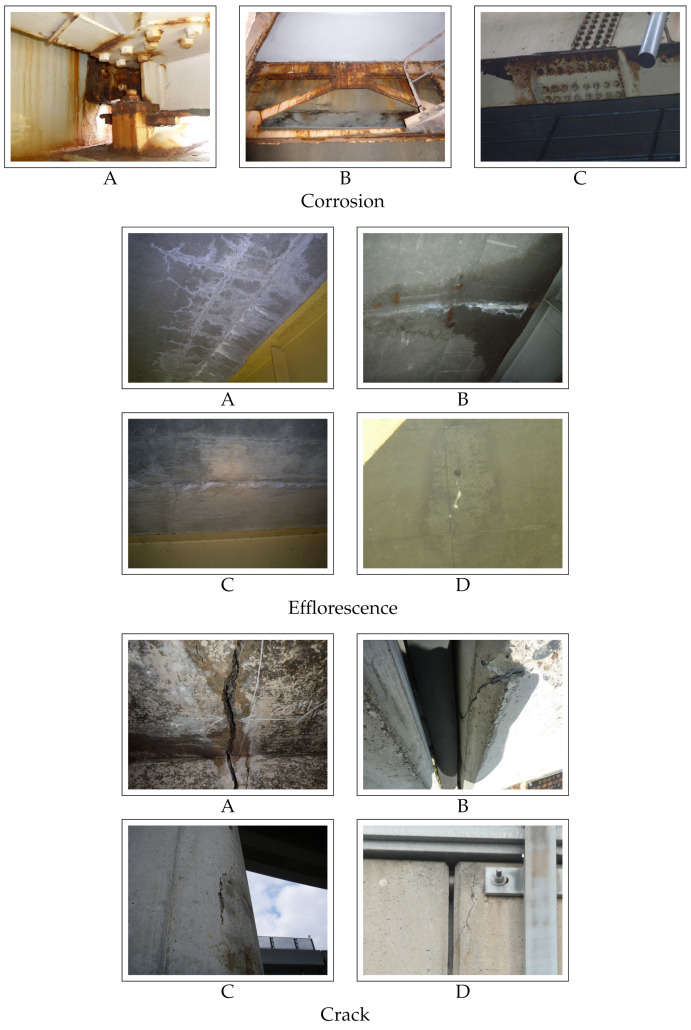
Examples of each type of distress with different levels.

In the experiment, we used three datasets consisting of distress images of road infrastructure provided by East Nippon Expressway Company Limited. These datasets consist of images of corrosion, efflorescence, and crack. They are the most important distresses and are often used in previous studies [23,24,25,26]. The deterioration levels of corrosion to be estimated were “A”, “B”, and “C” in descending order of the risk. Moreover, the deterioration levels of efflorescence and crack to be estimated were “A”, “B”, “C”, and “D”. These deterioration levels are explained in detail in [27]. Examples of each type of distresses are shown in Figure 3. The datasets of corrosion, efflorescence, and crack consist of 6865, 5184, and 9982 distress images, respectively. The detailed numbers of images used in training, validation, and test for each level are shown in Table 1, Table 2 and Table 3. The value of α in Equation (8) was experimentally set to 1. To evaluate the estimation performance, we used Accuracy and F-measure, which is the metric calculated as the harmonic mean of Recall and Precision defined as follows:

(9) $$\begin{array}{cc} \mathrm{Accuracy} & =\frac{\mathrm{Number}\;\mathrm{of}\;\mathrm{correctly}\;\mathrm{estimated}\;\mathrm{images}}{\mathrm{Number}\;\mathrm{of}\;\mathrm{all}\;\mathrm{images}}, \end{array}$$

(10) $$\begin{array}{cc} F\text{-}\mathrm{measure} & =\frac{2\times \mathrm{Recall}\times \mathrm{Precision}}{\mathrm{Recall}+\mathrm{Precision}}, \end{array}$$

where

(11) $$\begin{array}{cc} \mathrm{Recall} & =\frac{\mathrm{Number}\;\mathrm{of}\;\mathrm{correctly}\;\mathrm{estimated}\;\mathrm{images}\;\mathrm{in}\;\mathrm{each}\;\mathrm{level}}{\mathrm{Number}\;\mathrm{of}\;\mathrm{correct}\;\mathrm{images}\;\mathrm{in}\;\mathrm{each}\;\mathrm{level}}, \end{array}$$

(12) $$\begin{array}{cc} \mathrm{Precision} & =\frac{\mathrm{Number}\;\mathrm{of}\;\mathrm{correctly}\;\mathrm{estimated}\;\mathrm{images}\;\mathrm{in}\;\mathrm{each}\;\mathrm{level}}{\mathrm{Number}\;\mathrm{of}\;\mathrm{all}\;\mathrm{images}\;\mathrm{estimated}\;\mathrm{into}\;\mathrm{each}\;\mathrm{level}}. \end{array}$$

The larger the metrics, the higher the estimation performance. To evaluate the effectiveness of the proposed method, we adopted ABN [10], ResNet-50 [21], DenseNet-201 [28], Inception-v4 [29], EfficientNet-B5 [30], and SENet-154 [9] as comparative methods. Compared to ABN, the performance improvement using ConfABN verifies the effectiveness of considering the confidence in the attention map. Furthermore, the comparison between ConfABN and ResNet-50 [21], DenseNet-201 [28], Inception-v4 [29], EfficientNet-B5 [30], and SENet-154 [9] used in general image recognition [9,21,28,29,30] is conducted to confirm the effectiveness of ConfABN in estimating the deterioration level. The distress images were resized to 224 × 224 pixels when they were input into the models. The ConfABN and ABN models were developed based on ResNet-50 [21]. First, the feature extractor consists of the layers “conv_1”, “conv2_x”, “conv3_x”, and “conv4_x” defined in [21]. For the attention branch, the shallow part close to the input was “conv5_x” in [21]. The feature maps output from the shallow part were used to generate the attention map and estimate the deterioration level. Furthermore, for the perception branch, “conv5_x” in [21] was used for the shallow part. The final estimation results were calculated by passing the feature maps output from the shallow part of the perception branch through GAP and a fully connected layer. The detailed construction of ABN is presented in [10], and ConfABN had the same structure as ABN, except for the confidence-aware attention mechanism. The computations in the experiment were conducted on a computer with an Intel(R) Core(TM) i9-10980XE CPU @ 3.00 GHz, 128.0 GB of RAM, and a TITAN RTX GPU.

### 3.2. Performance Evaluation and Discussion

In this subsection, we demonstrate the performance of the proposed method for estimating the deterioration level by referring to the F-measure and the examples of the estimation results. We observe the distribution of confidence in the attention maps of the input distress images during the training and test phases. Quantitative evaluation, qualitative evaluation, and discussion about the distribution of confidence are shown in Section 3.2.1, Section 3.2.2 and Section 3.2.3, respectively. Finally, in Section 3.2.4, the limitation and future work of the proposed method are presented.

#### 3.2.1. Quantitative Evaluation

We quantitatively evaluate the effectiveness of the proposed method. Table 4, Table 5 and Table 6 present the F-measure of ConfABN and comparative methods in estimating the deterioration levels of corrosion, efflorescence, and crack, respectively. As shown in these tables, the proposed method achieved the largest F-measure for almost all items of the three distress types compared to the comparative methods used in general image recognition tasks. For instance, the better performance of ConfABN compared to ABN shows the effectiveness of the proposed method that considers the confidence in the attention map. One of the exceptions is that ABN achieves a larger F-measure than ConfABN in Level “B” of crack. However, the average of F-measure of ABN is smaller than that of ConfABN. Additionally, SENet-154 achieves a larger F-measure than ConfABN for Levels “B” and “C” in estimating the deterioration level of efflorescence. However, in Levels “A” and “D” of efflorescence, the F-measure of ABN is smaller than that of ConfABN. Finally, the average F-measure of ABN is lower than that of ConfABN. Furthermore, ConfABN is more accurate than ABN and SENet-154 in estimating Level A, which is the most dangerous and must not be estimated incorrectly. Furthermore, as shown in Table 7, since the accuracy of ConfABN is higher than those of the comparative methods, the use of ConfABN is justified. The results show that the proposed method is the most accurate regardless of the types of distress; thus, it can effectively estimate the deterioration level.

#### 3.2.2. Qualitative Evaluation

**Figure 4:**
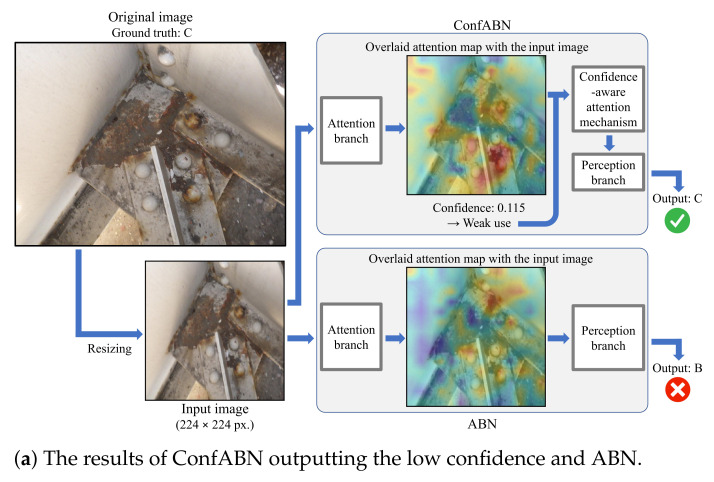

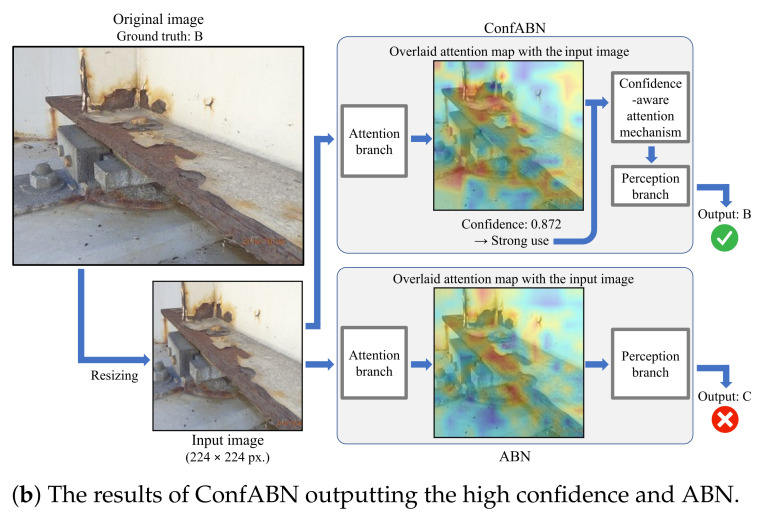
Examples of the estimation results using ConfABN and ABN for the images of corrosion.

**Figure 5:**
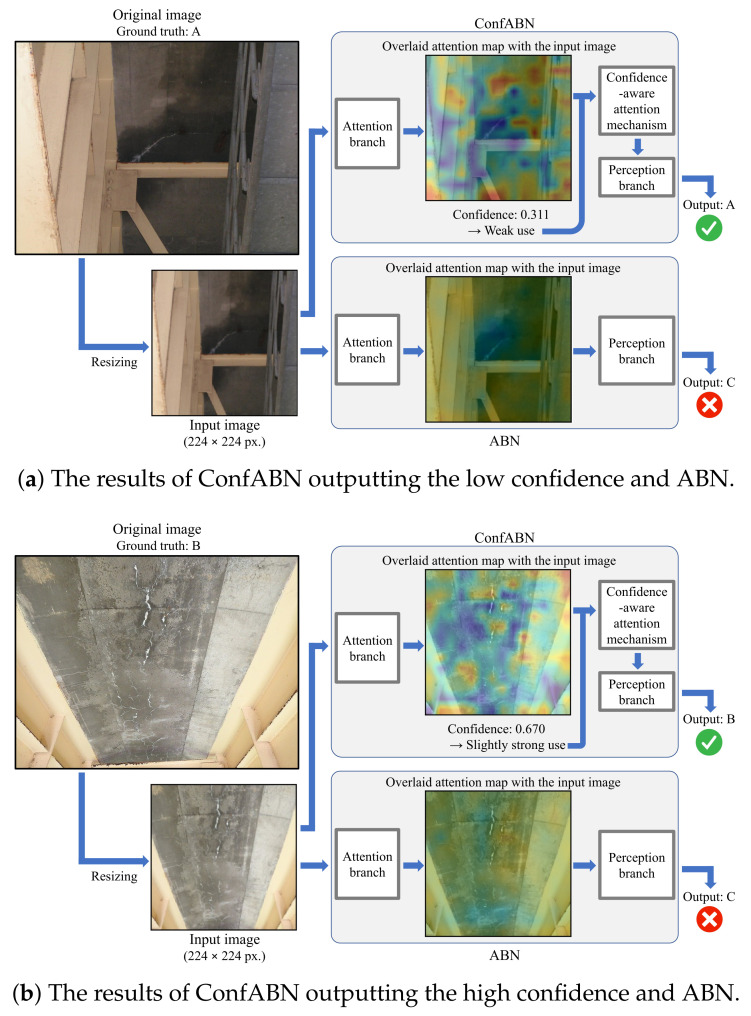
Examples of the estimation results using ConfABN and ABN for the images of efflorescence.

**Figure 6:**
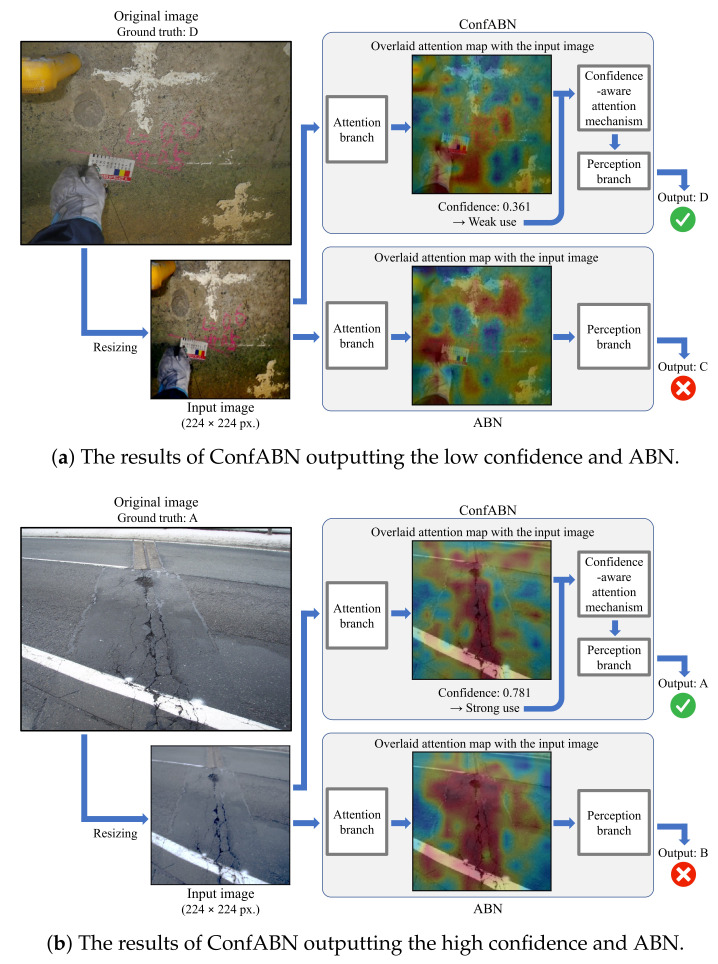
Examples of the estimation results using ConfABN and ABN for the images of a crack.

We qualitatively evaluate the effectiveness of ConfABN by comparing the estimation results of ConfABN with those of ABN for test images. Figure 4, Figure 5 and Figure 6 show examples of the estimation results of the deterioration levels of corrosion, efflorescence, and crack images, respectively. In the attention map, red indicates regions that received attention, and blue indicates regions that did not receive attention.

First, we discuss the examples of the estimation results of corrosion in Figure 4. Figure 4a shows the attention maps of ConfABN and ABN for estimating the deterioration level of the corrosion image with the ground truth of “C”, the confidence in the attention map of ConfABN, and the estimation results of ConfABN and ABN. In the input image, corrosion can be seen in the brown regions from the center to the top. However, since the attention maps of ConfABN and ABN highlight only a part of the corrosion region, they are ineffective. ConfABN estimates the low confidence in the attention map and reduces its influence. Then, it can output the correct estimation result. However, since ABN cannot consider the confidence in the attention map, it uses the ineffective attention map for the estimation and outputs the incorrect estimation result. Figure 4b shows an example of estimating the deterioration level of a corrosion image with the ground truth of “B”. In the input image, corrosion can be seen in the brown regions of the plate from the upper left to the lower right. Further, it can be seen in the brown regions of the root of the metal base. Since the attention map of ConfABN highlights the entire regions of corrosion, the attention map is effective for estimating the deterioration level. The attention map is strongly used in the perception branch due to the high confidence in the attention map of ConfABN. Thus, ConfABN succeeds in outputting the correct deterioration level. In contrast, although the attention map of ABN highlights most of the corrosion regions, it does not highlight some regions of corrosion. Thus, ABN outputs the incorrect deterioration level.

Next, we discuss the examples of the estimation results of efflorescence in Figure 5. Figure 5a shows an example of estimating the deterioration level of an efflorescence image with the ground truth of “A”. In the input image, efflorescence is revealed as a white line-like part in the center. The regions highlighted by the attention map of ConfABN do not include efflorescence. The attention map of ABN is fuzzy, and the highlight on efflorescence is weak. Since ConfABN can reduce the influence of the ineffective attention map, the perception branch of ConfABN observed the entire image again without focusing on the regions unrelated to efflorescence, and finally output the correct estimation result. However, ABN outputs the incorrect estimation result by focusing on the regions unrelated to efflorescence highlighted by the ineffective attention map. Figure 5b shows another example with the ground truth of “B”. As shown in the original image, efflorescence can be seen vertically in the center of the image. The attention map of ConfABN successfully highlights the regions of efflorescence; it also highlights the unrelated regions. The attention map is useful for estimating the deterioration level since the regions highlighted by the attention map include almost all efflorescence regions. However, trusting such an attention map entirely may lead to an incorrect estimation result. Meanwhile, ConfABN can appropriately handle this situation. The confidence in the attention map is lower than 0.7, suggesting that ConfABN does not have strong confidence in the attention map, including regions unrelated to efflorescence. The confidence-aware attention mechanism highlights the feature maps slightly more strongly using the attention map, including efflorescence-related and unrelated regions, and ConfABN outputs the correct deterioration level.

Finally, we discuss the examples of the estimation results of the crack in Figure 6. Figure 6a shows an example of estimating the deterioration level of a crack image with the ground truth of “D”. Since the crack is small and thin, the attention maps of ConfABN and ABN fail to highlight the region of the crack. ConfABN achieves correct estimation by utilizing the low confidence 0.361 to reduce the effect of ineffective attention maps. Figure 6b shows an example of estimating the deterioration level of a crack image with the ground truth “A”. The cracks appear vertically from the center to the bottom of the input image. The attention map of ConfABN focuses on the region of the cracks, and the high confidence shows that the attention map should be strongly used. Consequently, ConfABN outputs the correct estimation result.

As shown in the above examples, ConfABN can utilize effective attention maps strongly, and ineffective attention maps weakly by considering the confidence. In this way, the confidence-aware attention mechanism improves the performance of the deterioration level estimation.

#### 3.2.3. Distribution of Confidence

**Figure 7:**
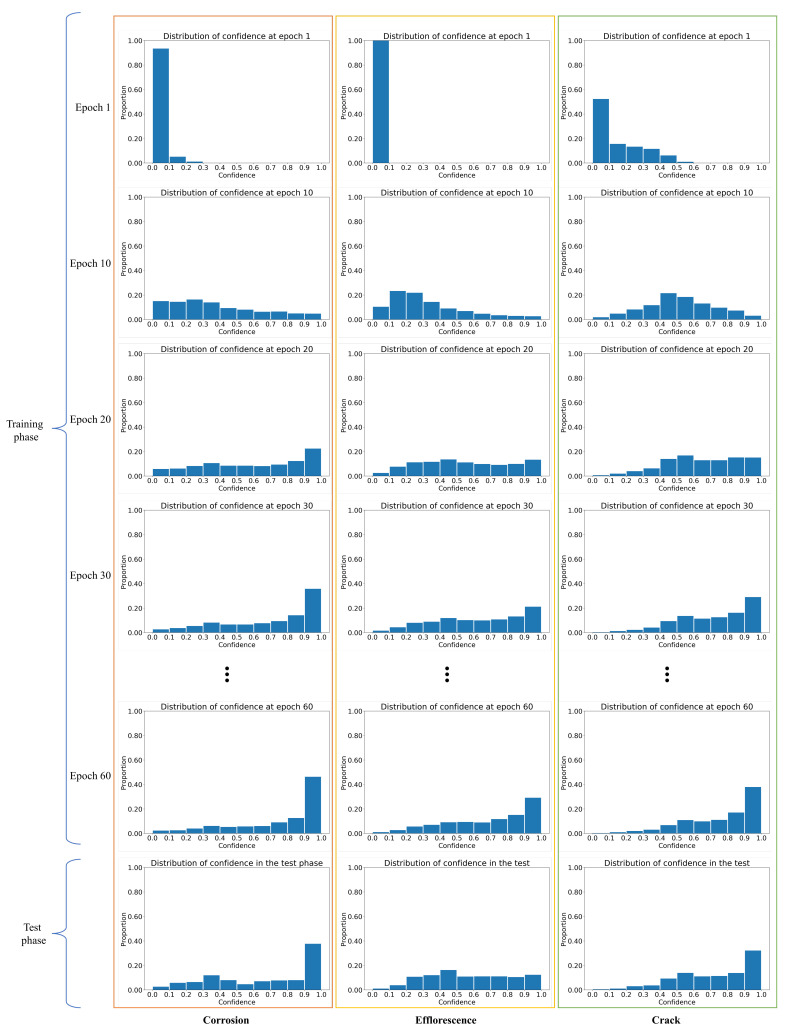
Distributions of the confidence in epochs 1, 10, 20, 30, and 60 of the training and test phases.

First, we describe the distribution of confidence during the training and test phases. Then, we discuss the effectiveness of using confidence in attention maps. Figure 7 shows the distributions of the confidence at the first, tenth, twentieth, thirtieth, and sixtieth epochs of the training and test phases for the three types of distresses. In epoch 1, i.e., at the beginning of the training phase, the confidence values in the attention maps are usually less than 0.1 for all types of distress. In the general ABN, the large influence of the attention map from the beginning of the training phase is likely to make the region of interest in the perception branch narrow and ineffective for the estimation. However, ConfABN solves this problem by weakening the influence of attention maps at the beginning of the training phase. Furthermore, the results in epochs 10, 20, 30, and 60 show a gradual increase in the high confidence in the attention maps as the training progresses. Although the results in epoch 60 show a high proportion of confidence close to 1.0 in the later training phase, there is still low confidence. The proportion of low confidence in the test phase is higher than in epoch 60. Such low confidence indicates the existence of ineffective attention maps even in the later training and test phases, indicating the effectiveness of considering confidence in such situations.

#### 3.2.4. Limitation and Future Work

We explain the limitation of the proposed method and our future work. Although the proposed method succeeds in reducing the influence of ineffective attention maps, we cannot directly generate effective attention maps, which is the limitation of the proposed method. One possible cause of the above problem is the resizing of the image when it is input into the model. For example, as shown in Figure 6b, resizing the original image makes it more difficult to find small or fine distresses. Consequently, the attention map fails to highlight the regions of distress in the feature maps. The advantages of the attention mechanism are not fully leveraged. Since we have already succeeded in reducing the effect of ineffective attention maps, our future work is to increase the number of effective attention maps by eliminating the process that makes it challenging to find the distresses. Furthermore, it is important to consider the relationship between crack and corrosion described in [31]. The task of this paper was only to estimate the deterioration levels when images of a single type of distress were input, and it is our future work to build a model that can consider the deterioration progression, relations of distresses, and situations where multiple distresses occur.

## 4. Conclusions

This paper proposed deterioration level estimation based on ConfABN that can control the influence of the attention map on feature maps according to the confidence. The proposed method solves the problem where ineffective attention maps are used when trying to highlight important regions in the feature maps. Specifically, the proposed method introduces a mechanism that calculates the confidence corresponding to an attention map based on entropy and utilizes effective attention maps strongly, and ineffective attention maps weakly. Consequently, it is possible to prevent regions unrelated to distresses from significantly influencing the estimation. Additionally, important regions in the feature maps can be highlighted to achieve more accurate estimation. The effectiveness of the proposed method has been confirmed by experiments using actual distress images for infrastructure inspection.

## Figures and Tables

**Table 1:** Numbers of the distress images of corrosion used in the experiment.

Deterioration Level	Training	Validation	Test
A	2178	147	155
B	1974	142	142
C	1816	157	154

**Table 2:** Numbers of the distress images of efflorescence used in the experiment.

Deterioration Level	Training	Validation	Test
A	1039	143	141
B	1125	150	152
C	1096	143	129
D	842	105	119

**Table 3:** Numbers of the distress images of crack used in the experiment.

Deterioration Level	Training	Validation	Test
A	1915	228	227
B	2085	237	265
C	1897	233	251
D	2102	265	277

**Table 4:** F-measure of ConfABN and comparative methods in estimating deterioration levels of corrosion.

	Level “A”	Level “B”	Level “C”	Average
ConfABN	**0.727**	**0.593**	**0.735**	**0.684**
ABN [10]	0.693	0.568	0.645	0.635
ResNet-50 [21]	0.614	0.544	0.660	0.606
DenseNet-201 [28]	0.609	0.529	0.651	0.597
Inception-v4 [29]	0.633	0.495	0.619	0.582
EfficientNet-B5 [30]	0.675	0.488	0.622	0.595
SENet-154 [9]	0.663	0.404	0.697	0.588

**Table 5:** F-measure of ConfABN and comparative methods in estimating deterioration levels of efflorescence.

	Level “A”	Level “B”	Level “C”	Level “D”	Average
ConfABN	**0.689**	0.563	0.433	**0.650**	**0.584**
ABN [10]	0.649	0.564	0.417	0.580	0.553
ResNet-50 [21]	0.598	0.492	0.427	0.632	0.537
DenseNet-201 [28]	0.651	0.431	0.421	0.598	0.525
Inception-v4 [29]	0.636	0.452	0.382	0.632	0.525
EfficientNet-B5 [30]	0.657	0.488	0.339	0.616	0.525
SENet-154 [9]	0.601	**0.583**	**0.448**	0.619	0.562

**Table 6:** F-measure of ConfABN and comparative methods in estimating deterioration levels of crack.

	Level “A”	Level “B”	Level “C”	Level “D”	Average
ConfABN	**0.778**	0.611	**0.558**	**0.687**	**0.658**
ABN [10]	0.752	**0.618**	0.553	0.676	0.649
ResNet-50 [21]	0.764	0.577	0.500	0.660	0.625
DenseNet-201 [28]	0.744	0.536	0.502	0.664	0.612
Inception-v4 [29]	0.726	0.571	0.480	0.652	0.607
EfficientNet-B5 [30]	0.749	0.572	0.497	0.647	0.616
SENet-154 [9]	0.717	0.609	0.472	0.673	0.618

**Table 7:** Accuracy of ConfABN and comparative methods in estimating deterioration levels of the three types of distress.

	Corrosion	Efflorescence	Crack
ConfABN	**0.690**	**0.586**	**0.658**
ABN [10]	0.634	0.551	0.649
ResNet-50 [21]	0.603	0.536	0.627
DenseNet-201 [28]	0.594	0.523	0.616
Inception-v4 [29]	0.581	0.529	0.611
EfficientNet-B5 [30]	0.599	0.527	0.618
SENet-154 [9]	0.612	0.560	0.623

## Data Availability

Experimental data cannot be disclosed.
